# Surgical treatment of gastric outlet obstruction from a large trichobezoar: A case report

**DOI:** 10.1016/j.ijscr.2019.04.002

**Published:** 2019-04-06

**Authors:** E. Chahine, R. Baghdady, N. El Kary, M. Dirani, M. Hayek, E. Saikaly, E. Chouillard

**Affiliations:** a Department of Minimally Invasive Surgery, Poissy Saint Germain Medical Center, Poissy, France; b Faculty of Medicine, Saint George Hospital University Medical Center, University of Balamand, Beirut, Lebanon

**Keywords:** Pica disorder, Bezoars, Gastric outlet obstruction, Case report

## Abstract

**Introduction:**

Bezoars are concretions of foreign indigestible material accumulating in the gastrointestinal tract leading to intraluminal mass formation that impairs the gastrointestinal motility and can lead to gastric obstruction of the small or the large bowel. There are different types of bezoars, named according to the material they are made of. These include phytobezoar, lactobezoar, pharmacobezoar, trichobezoar, and polybezoar. Trichobezoars (hair ball) are usually located in the stomach but may extend through the pylorus into the duodenum and small bowel (Rapunzel syndrome).

**Case presentation:**

Herein, we report a case of a young adult female known to have a long-standing trichophagia who presented with gastric outlet obstruction due to a large trichobezoar.

Endoscopy revealed a large and hard gastric trichobezoar not amenable to endoscopic retrieval leading to surgical extraction as a last resort.

**Discussion:**

They are almost always associated with trichotillomania and trichophagia or other psychiatric disorders. Trichobezoar can be treated either surgically by laparotomy/laparoscopy or by endoscopic intervention.

**Conclusion:**

Treatment should be coupled to psychiatric evaluation and therapy to prevent recurrence.

## Highlights

• Trichobezoar is a rare disorder that almost exclusively affects young females.
• We present a review of the main characteristics of this disease with a case of gastric outlet obstruction.
• A treatment should be coupled to psychiatric evaluation and therapy to prevent recurrence.

## 1 Introduction

Trichobezoars are generally seen in individuals with trichophagia, a psychiatric disorder, commonly seen in young adult females [1]. Trichobezoars are generally located in the stomach. However, if trichophagia is not reported early by the patient nor noticed by the guardians then it can develop into what is known as Rapunzel Syndrome, which is gastric trichobezoar extending into the small intestine. As trichophagia is the basis for trichobezoar formation, recurrence is inescapable if adequate psychiatric treatment and support are not provided after the surgical treatment.

Although not common, trichobezoars can result in devastating complications including death if left undetected [2].

Surgical intervention is often required in the management of large trichobezoars [3].

Herein, we present a case of a young female presenting with gastric outlet obstruction by large trichobezoar, diagnosed by cross-sectional imaging and treated surgically after failure of endoscopic intervention.

The work in this case has been reported in line with the SCARE criteria [4].

## 2 Case presentation

A14-year-old female patient known to have Pica disorder since the age of 2 years with a history of recurrent trichophagia was admitted to the emergency department for nausea, vomiting, and unintentional weight loss of 7 kg in 1 month.

On physical examination, the vital signs were stable, the patient appeared pale.

Abdominal exam revealed a large, firm and hard mass in the epigastric and left upper quadrant areas.

The result of the blood tests including complete blood count, electrolytes, BUN, creatinine, liver function tests, amylase and lipase were unremarkable.

**Fig. 1:**
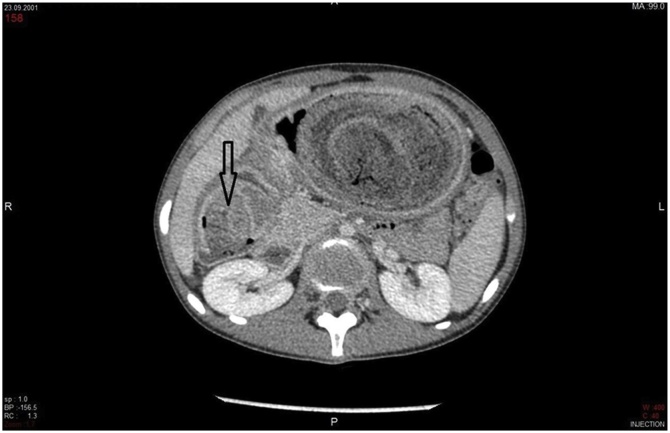
Contrast enhanced CT, sagittal view at the level of pyloric canal: passage of the large mass lesion is noted through the pyloric canal (Open arrow to the duodenum (D)).

**Fig. 2:**
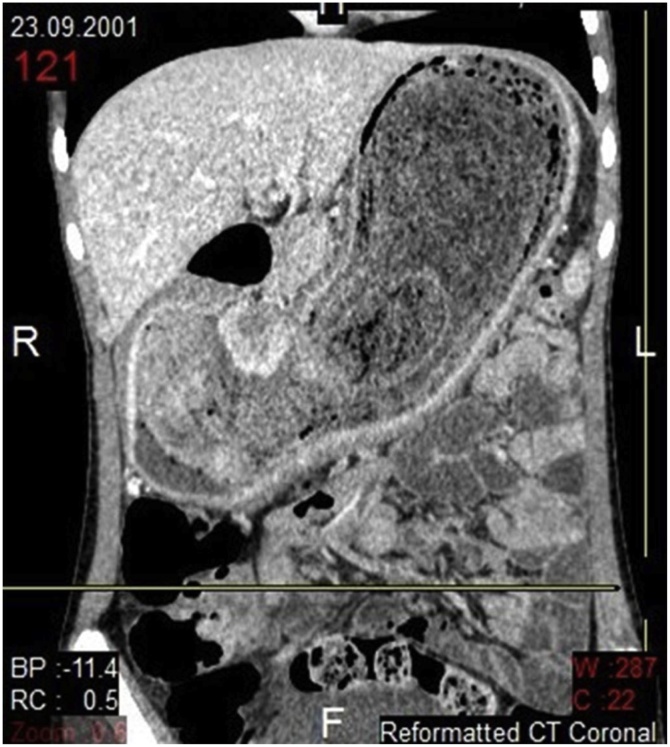
Contrast enhanced CT, Coronal view: Huge, well defined, multi-layered, heterogeneous, solid appearing, non-enhancing mass in the gastric lumen extending from the gastric fundus to the pyloric canal. Some of the layers of the mass are heterogeneously hyperdense. The mass lesion is separated from the gastric wall by gastric fluid. No evidence of abnormal gastric mural thickening.

The patient underwent a computed tomography (CT) of the abdomen and pelvis, revealing a large mass measuring 30 × 17 × 12 cm, well defined, multi-layered, heterogeneous, solid appearing, non-enhancing mass in the gastric lumen, extending from the gastric fundus to the pyloric canal. Some of the layers of this mass were heterogeneously hyperdense (Fig. 1). The lesion was separated from the gastric walls by gastric fluid. No evidence of abnormal gastric mural thickening was noted (Fig. 2).

Consequently, esophagogastroduodenoscopy (EGD) was done, revealing a collection of a large hard hairball occupying the entire lumen of the stomach from the fundus through the pylorus reaching the duodenum. Endoscopic intervention failed to retrieve the mass due to its large size and hard nature.

**Fig. 3:**
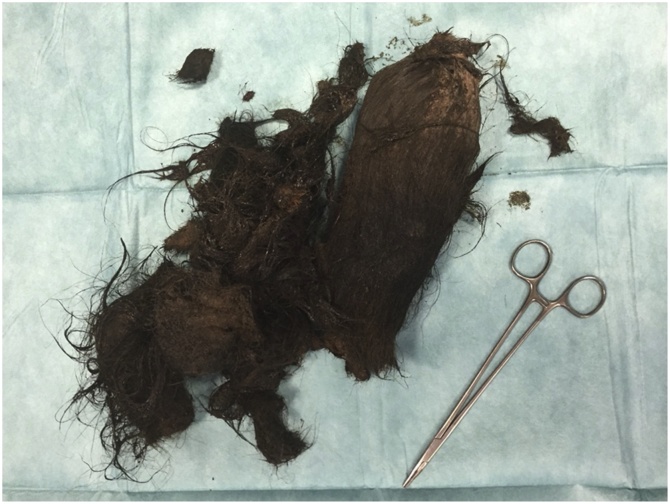
Huge, intra-luminal bezoar lesion removed from the stomach.

Subsequently, surgical intervention was planned through a midline laparotomy. A large solid fixed mass was palpated in the stomach. A 7 cm longitudinal gastrotomy was done on the anterior gastric wall, 6 cm from the pylorus. A large trichobezoar, filling the entire stomach and the first portion of the duodenum, was identified and removed (Fig. 3). The gastrostomy site was repaired in two layers with continuous 3.0 PDS (polydioxanone Ethicon). The abdominal incision was closed in two layers with continuous Vicryl 1 suture and skin was closed with staples. The total operative time was 50 min and the weight of the bezoar was 8 kg. The postoperative course was uneventful and the patient was referred to behavioral and mental health providers.

## 3 Discussion

Trichobezoar is a rare disorder that almost exclusively affects young females. Most patients with trichobezoar suffer from psychiatric disorders including trichotillomania and trichophagia [1]. The site of hair pulling is most commonly from the scalp, but can occur from the eyelashes, eye-brows and pubic area [5,6]. Rarely patients with this disorder chew hair from other sources including hair from wigs [3,7].

Human hair is resistant to digestion as well as peristalsis due to its smooth surface, leading to its accumulation between the mucosal folds of the stomach. Over a period of time, continuous ingestion of hair leads to the impaction of hair together with mucus and food, causing the formation of a trichobezoar [8,9].

In most cases, the trichobezoar is confined within the stomach. However, in some cases, it extends through the pylorus into the jejunum, ileum or even colon. This condition is called Rapunzel syndrome, first described by Vaughan Jr. et al. in 1968 [10].

Affected patients remain asymptomatic for many years. Symptoms develop as the bezoar increases in size. The most common presentations are abdominal pain, nausea/vomiting, obstruction and peritonitis. Less often, the patients present with weight loss, anorexia, hematemesis and intussusception [1,10].

Other complications include gastric ulceration, obstructive jaundice, acute pancreatitis and gastric emphysema. In addition, malabsorption related complications include protein losing enteropathy, iron deficiency and megaloblastic anemia [11].

Different therapeutic options have been employed to treat this condition, including laparotomy, endoscopic removal and laparoscopic removal [1,5,12]. Conventional laparotomy is still the treatment of choice depending on the size and site of trichobezoar [3].

## 4 Conclusion

Physicians, surgeons and radiologists should consider trichobezoars among the differential diagnosis for young females with abdominal pain and presence of an upper abdominal mass. Endoscopic or surgical removal can be performed safely and effectively. Treatment should be coupled to psychiatric evaluation and therapy to prevent recurrence.

## Conflicts of interest

No potential conflict of interest relevant to this article was reported.

## Funding

There are no sources of funding for this research.

## Ethical approval

Not applicable. The study is exempt from ethical approval in our institution.

## Consent

Consent has been obtained from the patient mother’s on behalf of the patient. No identifying details have been used in the article.

## Author contribution

CHOUILLARD E – study concept, and final approval. CHAHINE E, EL KARY N, **DIRANI M, HAYEK M, SAIKALY E** – acquired and interpreted the data and drafted the manuscript with editing. CHAHINE E, BAGHDADY R. – performed the operation and perioperative management of the patient, revision of the manuscript. All authors read and approved the final manuscript.

## Registration of research studies

Not available.

## Guarantor

CHOUILLARD E, CHAHINE E.

## Provenance and peer review

Not commissioned, externally peer-reviewed
